# Umbrella review of systematic reviews on the efficacy and safety of using mesh in the prevention of parastomal hernias

**DOI:** 10.1007/s10029-024-03137-2

**Published:** 2024-08-23

**Authors:** Sameh Hany Emile, Justin Dourado, Peter Rogers, Anjelli Wignakumar, Nir Horesh, Zoe Garoufalia, Rachel Gefen, Steven D. Wexner

**Affiliations:** 1 Ellen Leifer Shulman and Steven Shulman Digestive Disease Center, Cleveland Clinic Florida, 2950 Cleveland Clinic Blvd, Weston, 33179 FL United States; 2 Colorectal Surgery Unit, General Surgery Department, Mansoura University Hospitals, Mansoura, Egypt; 3 Department of Surgery and Transplantation, Department of General Surgery, Sheba Medical Center, Ramat-Gan Jerusalem, Israel; 4 Department of General Surgery, Hadassah Medical Center and Faculty of Medicine, Hebrew University of Jerusalem, Jerusalem, Israel

**Keywords:** Mesh, Parastomal hernia, Efficacy, Safety, Umbrella, Systematic review

## Abstract

**Background:**

This umbrella review aimed to summarize the findings and conclusions of published systematic reviews on the prophylactic role of mesh against parastomal hernias in colorectal surgery.

**Methods:**

PRISMA-compliant umbrella overview of systematic reviews on the role of mesh in prevention of parastomal hernias was conducted. PubMed and Scopus were searched through November 2023. Main outcomes were efficacy and safety of mesh. Efficacy was assessed by the rates of clinically and radiologically detected hernias and the need for surgical repair, while safety was assessed by the rates of overall complications.

**Results:**

19 systematic reviews were assessed; 7 included only patients with end colostomy and 12 included patients with either ileostomy or colostomy. The use of mesh significantly reduced the risk of clinically detected parastomal hernias in all reviews except one. Seven reviews reported a significantly lower risk of radiologically detected parastomal hernias with the use of mesh. The pooled hazards ratio of clinically detected and radiologically detected parastomal hernias was 0.33 (95%CI: 0.26–0.41) and 0.55 (95%CI: 0.45–0.68), respectively. Six reviews reported a significant reduction in the need for surgical repair when a mesh was used whereas six reviews found a similar need for hernia repair. The pooled hazards ratio for surgical hernia repair was 0.46 (95%CI: 0.35–0.62). Eight reviews reported similar complications in the two groups. The pooled hazard ratio of complications was 0.81 (95%CI: 0.66-1).

**Conclusions:**

The use of surgical mesh is likely effective and safe in the prevention of parastomal hernias without an increased risk of overall complications.

**Supplementary Information:**

The online version contains supplementary material available at 10.1007/s10029-024-03137-2.

## Introduction

Creating intestinal stomas is a common practice for a variety of colorectal conditions, including colorectal cancer (CRC) and inflammatory bowel disease (IBD). It has been estimated that > 100,000 stomas are annually created in the USA [1]. Although previous research showed a decrease in the number of intestinal stomas in patients with CRC, they are still performed in many patients [2].

The type of intestinal stomas varies. Ileostomies and colostomies are the two main types of intestinal stomas, both of which can be either temporary or permanent. The method of stoma construction also varies as an end stoma is technically different from a loop stoma [3]. Stoma-related complications can affect 20–70% of patients with an ostomy [1]. Regardless of the type of stoma, most stomas share a common complication profile that encompasses stoma prolapse, retraction, stenosis, ischemia, and hernia [4]. Some complications are more pertinent to one stoma type than another. Dehydration, for instance, is more commonly encountered with high-output ileostomies whereas hernias are more common in colostomies [5].

Parastomal hernias are essentially incisional hernias that develop through the abdominal wall defect at the stoma site [6]. Parastomal hernias are a common event, particularly in patients with permanent stomas. While clinically significant parastomal hernias affect up to 39% of patients with a colostomy, the actual incidence may reach up to 80% if determined by cross-sectional imaging [6]. Treatment of parastomal hernias is typically challenging with pooled recurrence rates varying between 10.2% and 27.9% after laparoscopic repairs [7]. Therefore, prevention of parastomal hernias is essential to avoid the challenges associated with subsequent surgical repair. Various methods were devised to reduce the incidence of parastomal hernias. Maintaining an ostomy size of < 3 cm was suggested as an opening of > 3 cm is associated with a greater incidence of parastomal hernias [8]. Stoma location may play a significant role as placing it through the rectus muscle, rather than lateral to it, was recommended by one study [9] whereas another study showed that locating the stoma at the specimen extraction site may increase the risk of parastomal hernias [10]. Extra-peritoneal tunneling of the stoma may further reduce the risk of hernias [11]. Finally, reinforcing the stoma site with a mesh was suggested to reduce the incidence of parastomal hernias and, to this end, different types and locations of meshes were investigated in the literature. Several systematic reviews and meta-analyses have assessed the prophylactic role of meshes in preventing parastomal hernias. However, these systematic reviews searched different databases and used different selection criteria and analytic methods. This heterogeneity eventually may result in different and sometimes conflicting conclusions on the efficacy of mesh for parastomal hernia prevention. Therefore, we conducted the present umbrella review to overview and assess the findings and conclusions of published systematic reviews on the prophylactic role of mesh against parastomal hernias in colorectal surgery.

## Methods

### Search strategy

This study was an umbrella overview of systematic reviews that assessed the prophylactic role of mesh in the prevention of parastomal hernias. The current review was reported in adherence with the reporting guideline of the umbrella review approach [12], and compliant with the PRISMA 2020 guidelines [13]. Prospective registration of the protocol of this umbrella review in the prospective register of systematic reviews (PROSPERO) was done to avoid reporting bias (CRD42023486442).

Two authors (S.E. & J.D.) independently searched PubMed and Scopus from their inception through November 2023 for systematic reviews with quantitative meta-analyses on the efficacy and safety of using mesh to prevent parastomal hernias. The article screening process consisted of two steps: in the first step, title/abstract screening, non-eligible articles were excluded and in the second step, the full text of the remaining articles was reviewed by two authors.

MeSH terms used in the search were (Surgical mesh), (Ostomy), and (Hernia). The following keywords syntax was used in our search: [“Stoma” OR “Colostomy” OR “Ileostomy” OR “Ostomy”] AND [“Parastomal hernia” OR “Peristomal hernia” OR [Incisional hernia] AND [“Mesh” OR “Surgical Mesh”] AND [“Prophylaxis” OR “Prevention”] AND [“Systematic review” OR Meta-analysis”]. To maximize the sensitivity of the search and look for further eligible studies, we activated the “related articles” function of PubMed and manually screened the reference sections of the initially recovered studies.

### Inclusion criteria

This umbrella review included only systematic reviews that provided summary pooled estimates of at least one of the main outcomes of the review using odds ratio (OR), relative risk (RR), risk ratio (RR), or hazard ratio (HR). Systematic reviews that fulfilled the following PICO criteria were eligible for inclusion:

- P (population): Patients who underwent colorectal surgery and had an ostomy (ileostomy or colostomy).
- I (intervention): Using surgical mesh to reinforce the stoma site.
- C (comparator): Not using mesh.
- O (outcome): Parastomal hernia and complications.

We excluded non-systematic reviews such as narrative and scoping reviews, systematic reviews without meta-analyses of the outcomes, original articles, editorials, and experimental studies. Only reviews that had English language full-text available were included.

### Data collected

The following data were extracted from the studies by two authors into an Excel spreadsheet:

- Authors, study design and country of the authors.
- Databases searched in each systematic review.
- Number and type of studies included in each review.
- Number, age, sex, and body mass index (BMI) of patients in each review.
- Type of stoma and type of mesh in each review.
- Quality of original studies included in each review.
- Primary and secondary outcomes.

### Outcomes

The primary outcome of this umbrella review was the efficacy of mesh assessed by the rates of clinically and radiologically detected parastomal hernias and the need for surgical repair of the hernia. The secondary outcome was safety assessed by the rates of total complications, stomal necrosis, and stomal stenosis.

### Quality assessment

An independent assessment of the quality of each systematic review was conducted by three authors (J.D., P.R. & A.W.) using the AMSTAR-2 tool that consists of 16 questions. The overall confidence in the results of the review was rated as high, moderate, low, and critically low [14]. The certainty of evidence for each outcome was assessed using the GRADE approach and was graded as high, moderate, low, or critically low. Publication bias was assessed by visual inspection of the symmetry of funnel plots for each outcome.

### Statistical analysis

Data were analyzed using an open source, cross-platform meta-analysis software “openMeta[Analyst]™” version 12.11.14 and EZR (version 1.55) and R software (version 4.1.2). Continuous variables were expressed as mean ± standard deviation (SD), or median and normal range. Categorical variables were expressed as numbers and percentages. P values less than 0.05 were considered significant.

The effect estimates of parastomal hernias, surgical hernia repair, and complications in each systematic review were pooled and the pooled hazard ratios of each outcome were calculated using the generic inverse variance approach. Statistical heterogeneity was assessed by the p-value of the Inconsistency (I^2^) statistics. Heterogeneity was considered low if I^2^ < 25% and high if I^2^ > 75%. A fixed-effect model was used to pool data if no significant statistical heterogeneity was present, and the random-effect model was used if significant heterogeneity was detected. To assess the extent of overlap of the original studies included in each systematic review, we used the corrected covered area (CCA) index methodology for estimating pairwise overlap in umbrella reviews [15]. The following equation was used $\:\text{C}\text{C}\text{A}=\text{N}-\text{r}/\left(\text{r}\:\text{*}\:\text{c}\right)-\text{r},$ where N is the number of times primary publications were cited in the reviews, including double-counting, r is the number of unique primary publications that were cited only in one review, and c is the number of systematic reviews included. Overlap was considered slight as the CCA was 0–5% moderate if the CCA was 6–10%, and high if the CCA was 11–15% while a CCA > 15% signified a very high overlap.

## Results

### Characteristics of the studies

**Fig. 1:**
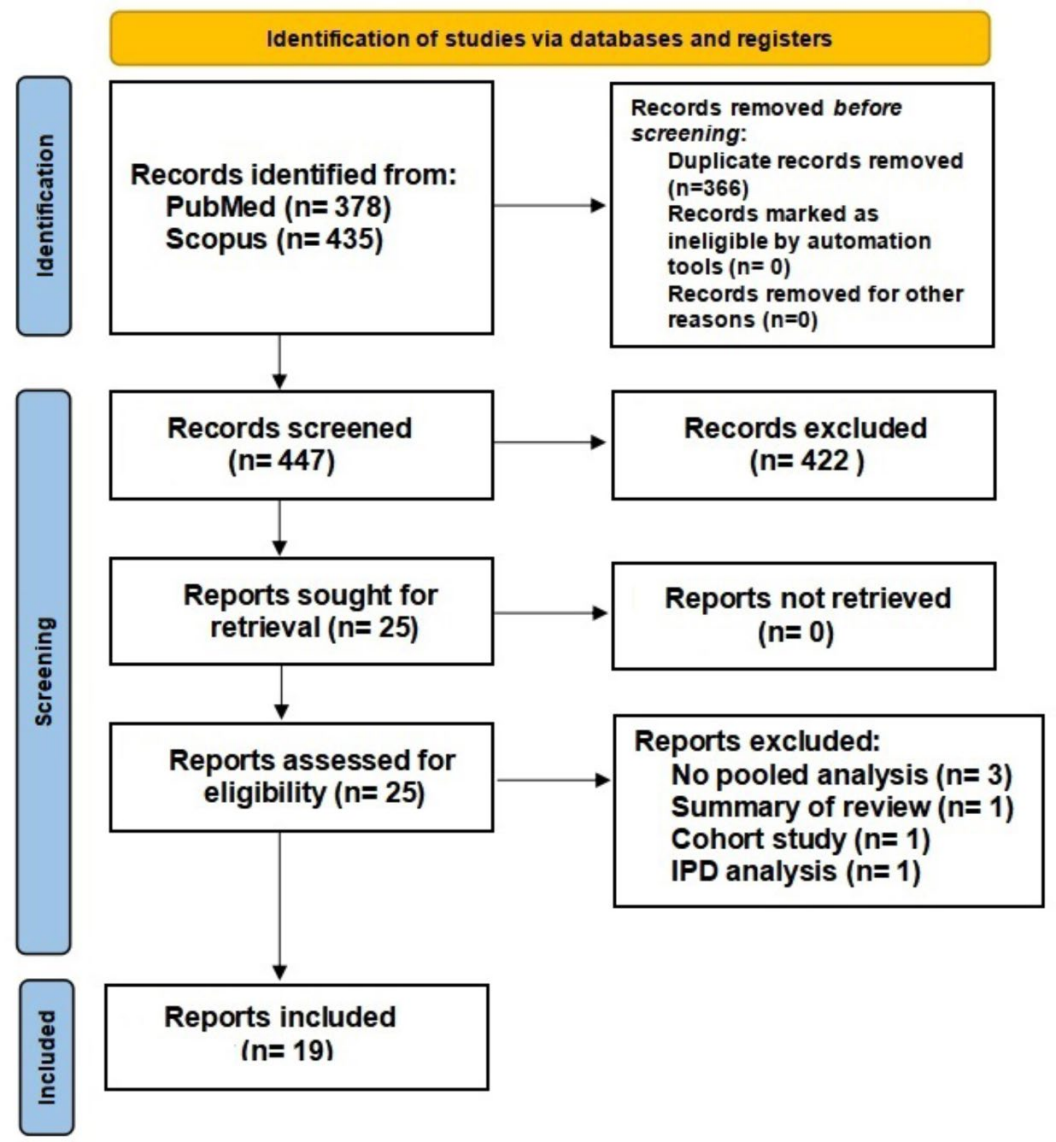
PRISMA flow chart

After screening 447 records, 19 systematic reviews with meta-analyses [16–34] were included (Fig. 1). The reviews were published between 2010 and 2023 and were emanated from the UK (*n* = 8), Europe (*n* = 4), Asia (*n* = 3), Canada (*n* = 2), Australia (*n* = 1), and New Zealand (*n* = 1).

The number of included reviews ranged between 3 and 13 across the systematic reviews and the number of patients ranged between 128 and 1252. All systematic reviews included only randomized controlled trials, except two reviews [22, 33] that included both randomized and observational studies. The median follow-up was at least 12 months in all reviews, and 7 reviews had a median follow-up of at least 24 months. The characteristics of the systematic reviews and the databases searched in each review are summarized in Table 1. The selection criteria, databases searched, and software used for the meta-analyses varied among the studies as shown in Appendix Table 1.

**Table 1:** Characteristics of the studies included

Study	Country	Number of studies	Number of patients	Type of studies	Follow-up of studies
**Verdaguer-Tremolosa et al.**,** 2023**	Spain	8	537	RCTs	Median 42 months (30.1–104.2)
**McKechnie et al.**,** 2022**	Canada	12	1252	RCTs	NR
**Mohiuddin et al.**,** 2021**	UK	13	1217	RCTs	Median 18 months (12–60)
**Sahebally et al.**,** 2021**	Australia	11	1097	RCTs	Median 26 months (12-65.2)
**Prudhomme et al.**,** 2021**	France	8	1038	RCTs	12 months
**Jones et al.**,** 2018**	UK	10	844	RCTs	12 months
**Findlay et al.**,** 2018**	UK	11	907	RCTs	12 months in 7 studies and 24 months in 2 studies.
**Pianka et al.**,** 2017**	Germany	11	755	RCTs and observational	Median 20 months (6.5–40)
**Cross et al.**,** 2017**	New Zealand	10	649	RCTs	Median 24 months (12–60)
**López-Cano et al.**,** 2017**	Spain	7	452	RCTs	Median 12 months (12-65.2)
**Patel et al.**,** 2017**	Canada	9	569	RCTs	Median 13 months (12–60)
**Chapman et al.**,** 2017**	UK	7	432	RCTs	12 months
**Cornille et al.**,** 2017**	UK	8	430	RCTs	Median 24 months (6.5–65)
**Wang et al.**,** 2016**	China	6	309	RCTs	Median 18 months (10.6–48)
**Zhu et al.**,** 2016**	China	8	522	RCTs	Median 20 months (3–60)
**Sajid et al.**,** 2012**	UK	3	128	RCTs	Median 29 months (12–60)
**Shabbir et al.**,** 2012**	UK	3	128	RCTs	Median 29 months (6.5–60)
**Wijeyekoon et al.**,** 2010**	UK	3	129	RCTs	Median 13 months (12–57)
**Tam et al.**,** 2010**	Taiwan	7	255	RCTs and observational	Median 29 months (6.5–65)

RCTs, randomized control trials; UK, United Kingdom; NR, not reported

The median age of patients ranged from 64.9 to 67.9 years and the median BMI ranged from 25.5 to 26.8 kg/m^2^. Male patients accounted for 55.3–75.2% of the population in the systematic reviews. Seven systematic reviews included only patients with end colostomy and 12 reviews included patients with either ileostomy or colostomy. Synthetic, composite, or biologic meshes were used in all reviews, except for two reviews [19, 29] that exclusively included synthetic meshes. The position of mesh placement was either sublay, intraperitoneal, or preperitoneal (Table 2).

**Table 2:** Characteristics of patients, stomas, and mesh used in the studies

Study	Age	Male	BMI	Type of stoma	Mesh position
**Verdaguer-Tremolosa et al.**,** 2023**	67	75.20%	26.3	End colostomy	Intraperitoneal-sublay
**McKechnie et al.**,** 2022**	66.7	62.70%	26.1	End colostomy	Preperitoneal-intraperitoneal-sublay
**Mohiuddin et al.**,** 2021**	43–72	NR	24.6–26.8	Colostomy or ileostomy	Preperitoneal-intraperitoneal-sublay
**Sahebally et al.**,** 2021**	67.9	60%	26.1	End colostomy	Intraperitoneal-sublay
**Prudhomme et al.**,** 2021**	NR	NR	NR	End colostomy	Intraperitoneal-sublay
**Jones et al.**,** 2018**	NR	NR	NR	Colostomy or ileostomy	Intraperitoneal-sublay
**Findlay et al.**,** 2018**	NR	NR	NR	Colostomy or ileostomy	NA
**Pianka et al.**,** 2017**	64.9	58.60%	26.5	Colostomy or ileostomy	Preperitoneal-intraperitoneal-sublay
**Cross et al.**,** 2017**	65.5	55.30%	26.3	Colostomy or ileostomy	Preperitoneal-intraperitoneal-sublay
**López-Cano et al.**,** 2017**	NR	NR	NR	End colostomy	Intraperitoneal-sublay
**Patel et al.**,** 2017**	NR	NR	NR	Colostomy or ileostomy	Preperitoneal-intraperitoneal-sublay
**Chapman et al.**,** 2017**	67.5	NR	26	Colostomy or ileostomy	Intraperitoneal-sublay
**Cornille et al.**,** 2017**	62.6	NR	26.6	Colostomy or ileostomy	Intraperitoneal-sublay
**Wang et al.**,** 2016**	67.5	NR	25.5	End colostomy	Intraperitoneal-sublay
**Zhu et al.**,** 2016**	67.1	NR	26.3	Colostomy	Intraperitoneal-sublay
**Sajid et al.**,** 2012**	67.5	57%	26	Colostomy or ileostomy	Preperitoneal-sublay
**Shabbir et al.**,** 2012**	60	57%	26	Colostomy or ileostomy	Intraperitoneal-sublay
**Wijeyekoon et al.**,** 2010**	67.5	NR	26	Colostomy or ileostomy	Preperitoneal-sublay
**Tam et al.**,** 2010**	NR	NR	NR	Colostomy or ileostomy	Intraperitoneal-sublay-onlay

BMI, body mass index; NR, not reported; NA, not available

### Efficacy

#### Clinical detected parastomal hernias

All systematic reviews except one that included patients with end colostomy [19] concluded that the use of mesh significantly reduced the risk of clinically detected parastomal hernias. The level of statistical heterogeneity was moderate-to-high. One review [23] that included RCTs and observational studies reported that mesh was protective against hernias in RCTs-only analysis, whereas the same conclusion was not confirmed on analysis of observational studies only (Table 3).

**Table 3:** Pooled effect estimates of efficacy of mesh in prevention of parastomal hernias

Study	Clinically detected hernia	Radiologically detected hernia	Surgical repair of hernia
**Verdaguer-Tremolosa et al.**,** 2023**	RR = 0.46 (95%CI: 0.23–0.95, *p* = 0.03, I2 = 80%)	RR = 0.75 (95%CI: 0.53–1.08, *p* = 0.12, I2 = 75%)	RR = 0.90 (95%CI: 0.51–1.56, *p* = 0.70, I^2^ = 0%)
**McKechnie et al.**,** 2022**	OR = 0.6 (95%CI: 0.46–0.8, *p* = 0.0003, I2 = 74%)	NR	NR
**Mohiuddin et al.**,** 2021**	RR = 0.65 (95%CI: 0.48–0.89, *p* = 0.01, I2 = 56%)	NR	RR = 0.63 (95%: 0.35–1.14, *p* = 0.39, I^2^ = 6)
**Sahebally et al.**,** 2021**	OR = 0.27 (95% CI: 0.12–0.61, *p* = 0.002, I2 = 75%)	OR = 0.39 (95%CI: 0.24–0.65, *p* = 0.0002; I2 = 49%)	OR = 0.54 (95%CI: 0.22–1.33, *p* = 0.18; I^2^ = 0)
**Prudhomme et al.**,** 2021**	RR = 0.65 (95% CI, 0.38; 1.13, *p* = 0.07, I2 = 58%)	RR = 0.69 (95% CI, 0.44; 1.10, *p* = 0.12, I2 = 48%)	RR = 0.38 (95%CI: 0.15, 0.99, *p* = 0.05, I^2^ = 0)
**Jones et al.**,** 2018**	RR = 0.53 (95% CI: 0.43–0.66, I2 = 69%)	NR	RR = 0.90 (95%CI: 0.50–1.64, I^2^ = 0)
**Findlay et al.**,** 2018**	OR = 0.23 (95%CI: 0.11–0.51; *p* = 0.0003, I2 = 66%)	OR = 0.43 (95%CI: 0.26–0.71; *p* = 0.001, I2 = 49%)	OR = 0.37 (95%CI: 0.24–0.55, *p* < 0.0001, I^2^ = 32%)
**Pianka et al.**,** 2017**	RCTs: OR = 0.24 (95%CI: 0.10–0.58; *p* = 0.034; I2 = 53.8%)- nRCT: OR = 0.59 (95%CI: 0.20–1.71; *p* = 0.13; I2 = 49.7%)	NR	NR
**Cross et al.**,** 2017**	OR = 0.24 (95%CI: 0.12–0.5, *p* < 0.001, I2 = 59%)	NR	NR
**López-Cano et al.**,** 2017**	RR = 0.24 (95%CI: 0.14–0.40; *p* < 0.0001, I2 = 6%)	RR = 0.61 (95%CI: 0.43–0.87; *p* = 0.006, I2 = 37%)	RR = 0.28 (95%CI: 0.10–0.78; *p* = 0.01, I^2^ = 0)
**Patel et al.**,** 2017**	OR = 0.21 (95%CI: 0.05–0.83, *p* = 0.03, I2 = 65%)	OR = 0.21 (95%CI: 0.11–0.38, *p* < 0.0001, I2 = 0)	OR = 0.36 (95%CI: 0.15–0.87, *p* = 0.02, I^2^ = 0)
**Chapman et al.**,** 2017**	RR = 0.34 (95%CI: 0.18–0.65, *p* = 0.001, I2 = 39%)	RR = 0.61 (95%CI: 0.42–0.89, *p* = 0.01, I2 = 44%)	OR = 0.30 (95%CI: 0.13–0.69, *p* = 0.005, I^2^ = 0)
**Cornille et al.**,** 2017**	RR = 0.23 (95%CI: 0.13–0.43, *p* < 0.0001, I2 = 15%)	RR = 0.68 (95%CI: 0.52–0.90, *p* = 0.006, I2 = 0)	NR
**Wang et al.**,** 2016**	RR = 0.42 (95%CI: 0.22–0.82, *p* = 0.01, I2 = 71%)	NR	RR = 0.23 (95%CI: 0.06–0.89, *p* = 0.03, I^2^ = 0)
**Zhu et al.**,** 2016**	RR = 0.22 (95%CI: 0.13–0.38, *p* < 0.0001,I2 = 0)	RR = 0.62 (95%CI: 0.47–0.82, *p* = 0.0008,I2 = 44%)	RR = 0.34 (95%CI: 0.14, 0.83, *p* = 0.02, I2 = 0)
**Sajid et al.**,** 2012**	OR = 0.11 (95%CI: 0.05–0.27, *p* < 0.0001, I2 = 57%)	NR	NR
**Shabbir et al.**,** 2012**	OR = 0.25 (95%CI: 0.13, 0.48, *p* < 0.0001, I2 = 36%)	NR	NR
**Wijeyekoon et al.**,** 2010**	RR = 0.23 (95%CI: 0.06–0.81; *p* = 0.02, I2 = 56%)	NR	RR = 0.13 (95%CI: 0.02–1.02, *p* = 0.05, I^2^ = 0)
**Tam et al.**,** 2010**	OR = 0.17 (95 CI: 0.07–0.40; *p* = 0.0001, I2 = 54%)	NR	NR

CI, confidence interval; RR, relative ratio; OR, odds ratio; NR, not reported

**Fig. 2:**
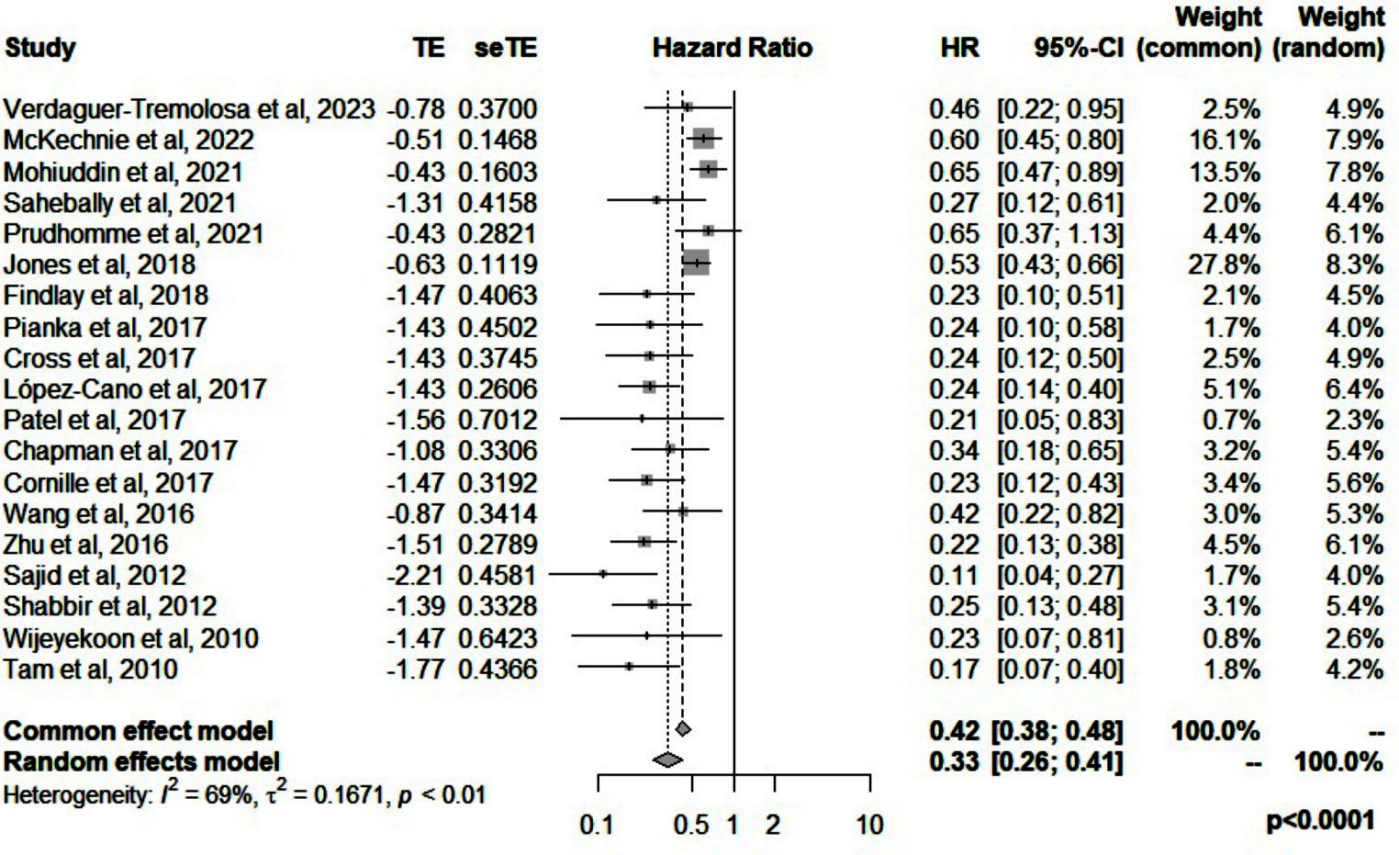
Forest plot for clinically detected hernia when mesh is used for prevention of parastomal hernias

The relative risk reduction of parastomal hernias with the use of mesh ranged between 35% and 89% with a median of 76%. When classified by the type of stoma, the use of mesh had a median risk reduction of 58% in reviews that included end colostomy only compared to 77% in reviews that included ileostomy and colostomy. The pooled hazards ratio of clinically detected parastomal hernias in the mesh group was 0.33 (95%CI: 0.26–0.41, *p* < 0.0001, I^2^ = 69%) (Fig. 2).

### Radiologically detected parastomal hernias

**Fig. 3:**
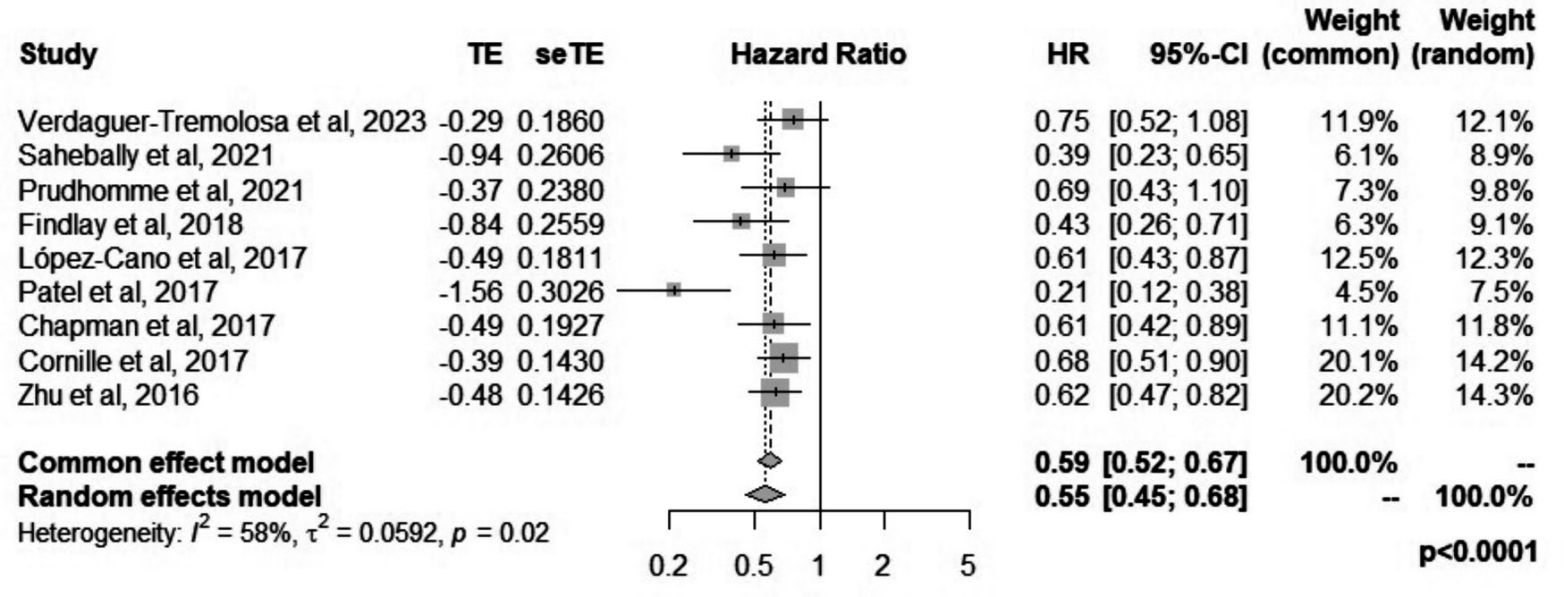
Forest plot for radiologically detected hernia when mesh is used for prevention of parastomal hernias

Nine reviews reported the pooled effect estimates of radiologically detected parastomal hernias. All except two reviews [16, 20] reported a significantly lower risk of radiologically detected parastomal hernias with the use of mesh. The relative risk reduction of radiologically detected parastomal hernias with the use of a mesh ranged between 25% and 79% with a median of 39%. When classified by the type of stoma, the median risk reduction of hernia with the use of mesh was 38% in reviews that included end colostomy only compared to 48% in reviews that included ileostomy and colostomy. The pooled hazards ratio of radiologically detected parastomal hernias in the mesh group was 0.55 (95%CI: 0.45–0.68, *p* < 0.0001, I^2^ = 58%) (Fig. 3).

### Need for surgical repair of hernia

**Fig. 4:**
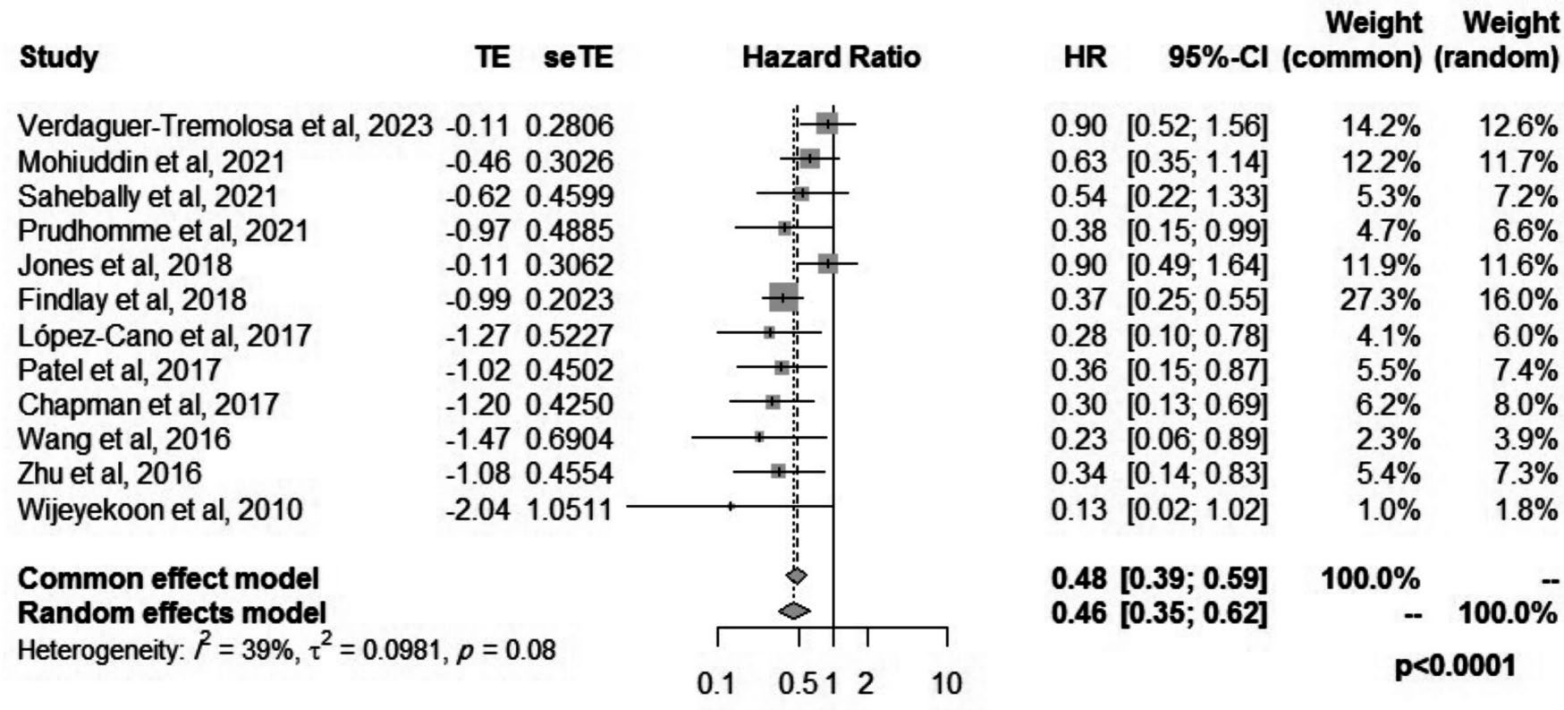
Forest plot for surgical repair of hernia when mesh is used for prevention of parastomal hernias

Twelve studies reported the pooled effect estimates for the need of surgical repair of parastomal hernias. Six reviews reported a significant reduction in the need for parastomal hernia repair when a mesh was used, whereas six reviews found a similar need for hernia repair between the two groups. The pooled hazards ratio for surgical hernia repair was 0.46 (95%CI: 0.35–0.62, *p* < 0.0001, I^2^ = 39%) (Fig. 4).

### Safety

**Fig. 5:**
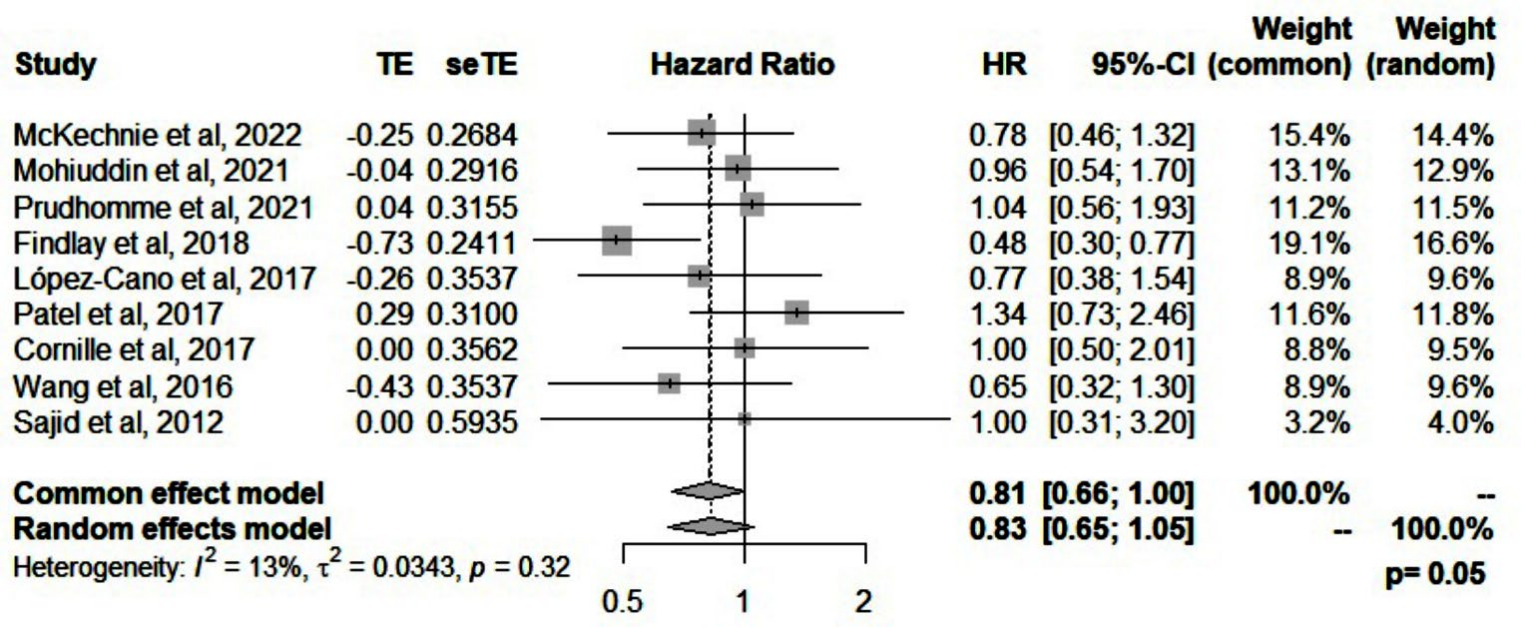
Forest plot for complications when mesh is used for prevention of parastomal hernias

Nine systematic reviews reported the pooled effect estimate of total complications; all except one review reported a similar risk of complications between the two groups. The statistical heterogeneity was low in all reviews except for one [17]. Only one review [21] reported lower odds of complications in favor of using a mesh (OR = 0.48, *p* = 0.002). The pooled hazard ratio of complications across all reviews was 0.81 (95%CI: 0.66-1, *p* = 0.05, I^2^ = 13%) (Fig. 5**)**. Stomal necrosis and stenosis were assessed in six and seven reviews, respectively. All reviews reported a similar risk of stomal necrosis and stenosis with mesh reinforcement to the control group. (Table 4)

**Table 4:** Pooled effect estimates of safety of mesh in prevention of parastomal hernias

Study	Complications	Stomal stenosis	Stomal necrosis
**Verdaguer-Tremolosa et al.**,** 2023**	NR	NR	NR
**McKechnie et al.**,** 2022**	OR = 0.78 (95%CI: 0.47–1.32, *p* = 0.36, I2 = 71%)	OR = 2.69 (95%C: I 0.63–4.55, *p* = 0.3, I2 = 0)	OR = 0.85 (95%CI: 0.39–1.89, *p* = 0.69, I2 = 0%)
**Mohiuddin et al.**,** 2021**	RR = 0.96 (95%CI: 0.55–1.7, *p* = 0.71, I2 = 0)	NR	NR
**Sahebally et al.**,** 2021**	NR	OR = 1.21 (95%CI: 0.41–3.53, *p* = 0.73, I2 = 0)	OR = 0.72, (95%CI = 0.29–1.8, *p* = 0.48, I2 = 0)
**Prudhomme et al.**,** 2021**	RR = 1.04 (95%CI: 0.56–1.93, *p* = 0.91, I2 = 0)	NR	NR
**Jones et al.**,** 2018**	NR	NR	RR = 0.89 (95%CI: 0.32–2.5, I2 = 0)
**Findlay et al.**,** 2018**	OR = 0.48 (95%CI: 0.30–0.77, *p* = 0.002)	NR	NR
**Pianka et al.**,** 2017**	NR	OR = 1.75 (95%CI: 0.49–6.35, *p* = 0.91, I2 = 0)	0.65 (95% CI 0.25–1.71; *p* = 0.98; I2 = 0%)
**Cross et al.**,** 2017**	NR	OR = 3.7 (95%CI: 0.75–18.1, *p* = 0.11)	OR = 0.8 (95%CI:0.24–2.7, *p* = 0.73)
**López-Cano et al.**,** 2017**	RR = 0.77 (95%CI: 0.39–1.54; *p* = 0.46, I2 = 0)	NR	NR
**Patel et al.**,** 2017**	OR = 1.34 (95%CI:0.73–2.46, *p* = 0.34, I2 = 34%)	NR	NR
**Chapman et al.**,** 2017**	NR	OR = 2.41 (95%CI: 0.73–8.01, *p* = 0.15, I2 = 0)	NR
**Cornille et al.**,** 2017**	RR = 1.0 (95%CI: 0.49–2.01, *p* = 0.99, I2 = 0)	NR	NR
**Wang et al.**,** 2016**	RR = 0.65 (95%CI: 0.33–1.3, *p* = 0.23, I = 0)	NR	NR
**Zhu et al.**,** 2016**	NR	RR = 1.67 (95%CI: 0.36–7.75, *p* = 0.51, I2 = 0)	RR = 0.58 (95%CI: 0.22, 1.50, *p* = 0.26, I2 = 0)
**Sajid et al.**,** 2012**	OR = 1 (95%CI: 0.36–3.2, *p* = 1, I2 = 0)	NR	NR
**Shabbir et al.**,** 2012**	NR	NR	NR
**Wijeyekoon et al.**,** 2010**	NR	NR	NR
**Tam et al.**,** 2010**	NR	NR	NR

NR: Not reported; OR: Odds ratio; RR: Relative ratio; CI: Confidence interval

**Fig. 6:**
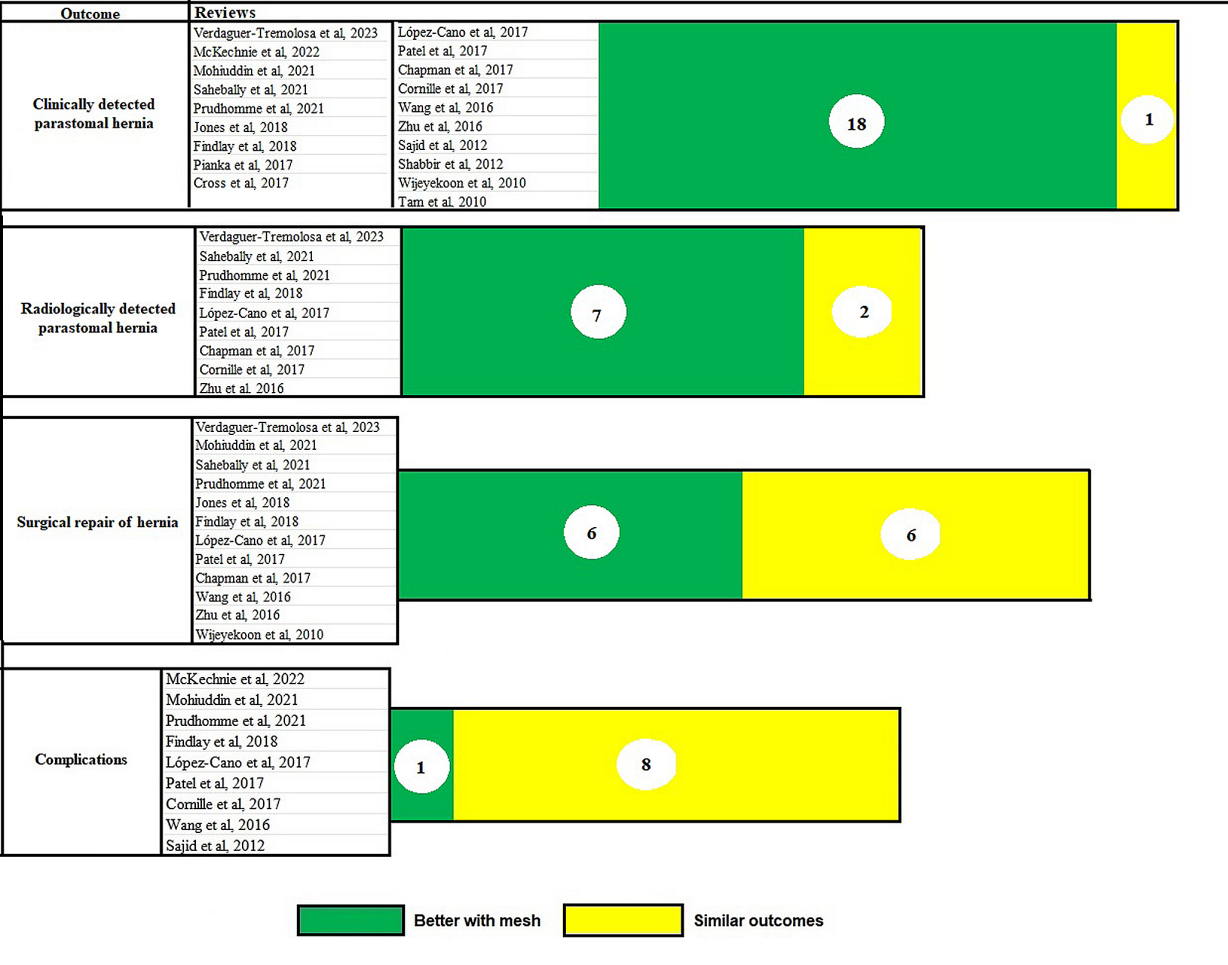
Visual representation of the conclusions of the published systematic reviews on the efficacy and safety of mesh in the prevention of parastomal hernia

A visual representation of the pooled efficacy and safety of mesh in parastomal hernia prevention is shown in Fig. 6.

### Subgroup analysis of studies with follow-up ≥ 24 months

A subgroup meta-analysis of the reviews that had a median follow-up of at least 24 months revealed that using prophylactic mesh was associated with lower hazards of clinically detected parastomal hernias (HR: 0.24; 95%CI: 0.18–0.32, *p* < 0.0001, I^2^ = 11%), and radiologically detected parastomal hernias (HR: 0.63; 95%CI: 0.53–0.76, *p* < 0.0001, I^2^ = 34%). However, the pooled hazard ratios for surgical hernia repair (HR: 0.78; 95%CI: 0.49–1.25, *p* = 0.308, I^2^ = 0) and complications (HR: 1.01; 95%CI: 0.66–1.56, *p* = 0.931, I^2^ = 0) were similar between the mesh and control groups.

### Quality of reviews and certainty of evidence

Overall, the majority of the reviews were of poor quality. Four reviews had moderate quality, 11 had low quality, and four had very low quality. The quality of each systematic review and the studies included within each review are shown in Appendix Table 2. All outcomes had a low certainty of evidence, except complications that had a moderate certainty (Appendix Table 3). The symmetry of the funnel plots for each outcome indicated the absence of publication bias (Supplementary figure). The CCA was 0.97%, indicating a slight overlap between the systematic reviews included in the pooled analysis.

## Discussion

Our overview of 19 systematic reviews showed that the use of a mesh to reinforce the stoma site was deemed effective in the prevention of parastomal hernias in almost all systematic reviews. However, while using a mesh significantly reduced the need for surgical repair of parastomal hernias in six reviews, another six reviews reported that the need for surgical repair was similar to the control group. The safety of using a mesh was established as all reviews that concluded that using a mesh did not increase the odds of complications compared to the control group.

The efficacy of mesh in the prevention of parastomal hernias is assessed by the rates of both clinically detected and radiologically detected hernias. The rate of parastomal hernia, whether detected clinically or radiologically by CT scanning, can reach up to 78% [35]. While the clinically detected hernias are usually those that warrant surgical correction to alleviate symptoms; sub-clinical parastomal hernias can afflict one-third of patients with stomas [36]. A study found a fair concordance between the clinical and radiologic assessment of parastomal hernias that increased with the increased size of the hernia sac. Thus the study recommended a combination of the two assessment methods [37].

Almost all systematic reviews agreed on the prophylactic role of mesh in an effort to prevent parastomal hernias that are both clinically and radiologically detected. Nonetheless, the pooled estimate of the reduction of clinically detected hernias was lower than that of radiologically detected hernias (0.33 vs. 0.55). This indicates that, while the use of a mesh can prevent clinically significant hernias by 67%, this preventative effect is reduced to 45% for sub-clinical hernias. The prevention of parastomal hernias with surgical meshes can be cost-effective by avoiding the cost of subsequent readmission and surgery to correct the hernia. Saha et al. [38] found that the use of prophylactic mesh served to decrease the rate of parastomal hernias from 21.5 to 7.5% with a mean difference in total costs of €2047, concluding that using a mesh was less costly and more effective.

It is important to highlight that two systematic reviews [16, 20], including the most recent review, did not support the prophylactic role of mesh in parastomal hernia prevention. These two reviews included only end colostomies that may partly explain the lack of a protective effect of the mesh. Our subgroup analyses also showed that the median reduction in the rate of parastomal hernias with end colostomy (58%) was less than that when ileostomies and colostomies were analyzed together (77%). This finding corroborates the notion that parastomal hernias may be more prevalent after a colostomy as compared to an ileostomy [39]. Another explanation of the lack of a protective effect of mesh in the most recent systematic review [16] is that it included only RCTs with long-term follow-up defined as > 24 months. It has been suggested that the rate of parastomal hernias tends to increase with time even with the use of a mesh and, thus, the true incidence essentially depends on the length of follow-up [40].

Another measure of the efficacy of mesh is the need for surgical repair of a parastomal hernia. Among 12 systematic reviews that assessed this parameter, only half reported that using a mesh conferred a significant reduction in the need for parastomal hernia repair. A pooled analysis of the 12 reviews showed that the use of a mesh can decrease the need for surgical hernia repair by 54%. This pooled estimate is midway between the pooled estimates of the prevention of clinical and radiological hernias (67% and 45%). This finding may imply that some sub-clinical hernias that were otherwise detected by CT scan only may have been subject to surgical repair, either in the setting of stoma revision or separately. A consensus on the management of parastomal hernia is hard to reach; nevertheless, the presence of a parastomal hernia per se does not warrant surgical correction. While symptomatic patients with a bulge or discomfort around the stoma and patients with recurrent partial intestinal obstruction may be indicated for surgical repair, patients with silent hernias detected via CT scan are usually treated with watchful waiting. Ultimately, the decision on surgical repair is usually tailored to each patient, weighing the potential risks of surgery against the estimated risk of hernia incarceration [41].

Almost all reviews concluded similar complication rates when mesh was used or not. The rates of stoma-specific complications such as stenosis or necrosis were not increased with the use of mesh. Only one systematic review by Findlay et al. [22] reported that using a mesh may reduce the odds of complications by 52%.

Some considerations should be made when deciding on the use of mesh for the prevention of parastomal hernias. The type of stoma and type of mesh are perhaps the most important considerations. While mesh placement can be reasonable in permanent stomas, such as end colostomies after non-restorative proctectomy, it may not be cost-effective in temporary stomas planned to be reversed within six months. The selection between synthetic and biologic mesh may also be challenging given the assumptions of a lower rate of complications with biologic mesh. However, as a systematic review concluded, while the routine use of synthetic mesh is cost-effective for the prevention of parastomal hernias, using a biological mesh may not be as cost-effective [22]. In a subgroup analysis, Patel et al. [26] also found that synthetic meshes reduced the odds of developing parastomal hernias, whereas this effect was not noted when biological meshes were used.

The present study is the first umbrella review that encompasses the published collective evidence on the role of mesh in preventing parastomal hernias. The review comprehensively assessed the efficacy and safety of mesh and provided a critical appraisal of the agreements and disagreements among the systematic reviews. Pooled analysis of the summary effect estimates of previous systematic reviews is often criticized because of the possible overlap and double counting of the original studies among the reviews. However, the overlap between the studies in our umbrella review was considered slight when assessed using an objective statistical method.

Some limitations of this review should be noted. Firstly, the poor quality of most included reviews signals a lower level of evidence. The heterogeneity in the type of stoma, type of mesh used, and follow-up duration among the systematic reviews and the original studies included within is another limitation that we tried to mitigate by conducting subgroup analyses. While all reviews had a median follow-up of at least 12 months, only 7 of 19 had a follow-up of *≥* 24. This observation indicates that most reviews reported only short-term outcomes of prophylactic mesh, whereas the literature attests to the importance of long follow-up to assess hernia outcomes. Nonetheless, a subgroup analysis of the few reviews that entailed longer follow-up durations confirmed the lower odds of parastomal hernias with the use of a prophylactic mesh. Another limitation to highlight is that most systematic reviews included the same studies and thus the same patients. To avoid such an overlap, we pooled the effect estimates of each review instead of the individual patients. Moreover, we used the CCA index to assess the extent of overlap of the original studies included in each systematic review, which indicated a slight overlap between the systematic reviews included in the pooled analysis.

## Conclusions

The use of surgical mesh is likely effective and safe in the prevention of parastomal hernias. However, the prophylactic effect of mesh may be lower in patients with end colostomy, who are typically at the highest risk for developing parastomal hernias. Longer follow-up of the published randomized trials is crucial to ascertain a sustained long-term prophylactic effect of the mesh. The addition of cost and additional operating room time and technical considerations may be obstacles to the adoption of prophylactic mesh placement.

## Electronic supplementary material

Below is the link to the electronic supplementary material.

Supplementary Material 1

Supplementary Material 2

Supplementary Material 3

Supplementary Material 4

Supplementary Material 5

Supplementary Material 6

Supplementary Material 7

Supplementary Material 8

Supplementary Material 9

Supplementary Material 10
