# Supplementary figures and images for: Umbrella review of systematic reviews on the efficacy and safety of using mesh in the prevention of parastomal hernias
Source: Hernia. 2024 Aug 23;28(5):1577–89. doi: 10.1007/s10029-024-03137-2 (PMC11457579; doi:10.1007/s10029-024-03137-2)

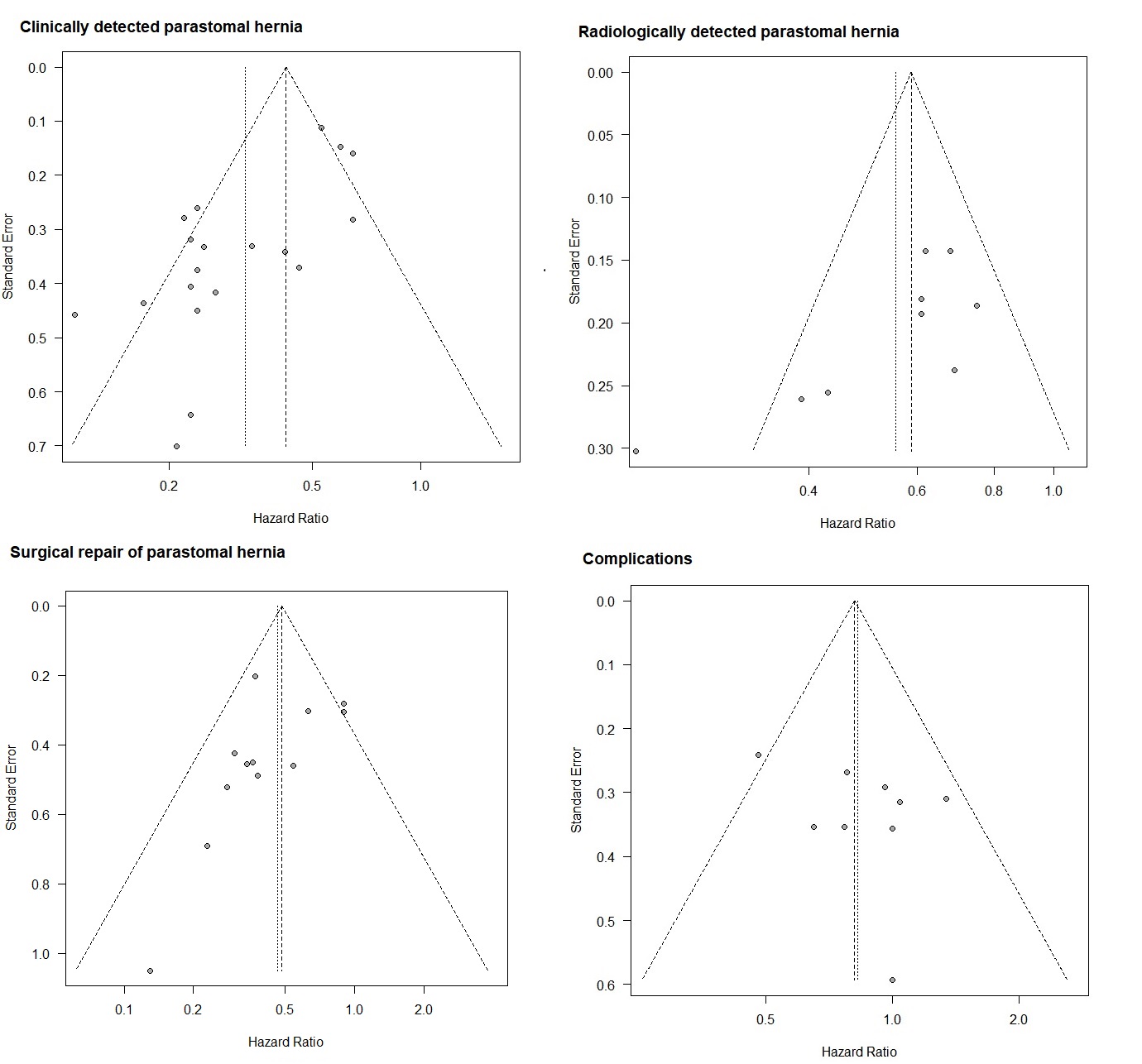

Supplement: Supplementary file 1 — Supplementary Material 1 [file 10029_2024_3137_MOESM1_ESM.jpg]
