# Supplementary material for: Umbrella review of systematic reviews on the efficacy and safety of using mesh in the prevention of parastomal hernias
Source: Hernia. 2024 Aug 23;28(5):1577–89. doi: 10.1007/s10029-024-03137-2 (PMC11457579; doi:10.1007/s10029-024-03137-2)
Supplement: Supplementary file 2 — Supplementary Material 2 [file 10029_2024_3137_MOESM2_ESM.docx]

**Appendix Table 1.** Selection criteria and databases searched in the reviews included

| **Study** | **Approach or mesh restrictions** | **Language restriction** | **Follow-up restrictions** | **Grey literature included** | **Database searched** | **Software used for the meta-analysis** |
| --- | --- | --- | --- | --- | --- | --- |
| **Verdaguer-Tremolosa et al, 2023** | No | Only articles in English and Spanish | No | No | MEDLINE, EMBASE, Cochrane Library, Web of Science, and Google Scholar | Cochrane Review Manager 5.4 (London, UK) and TSA software v0.9.5.10 beta (Copenhagen, Denmark) |
| **McKechnie et al, 2022** | Only elective surgery | No | No | Yes | MEDLINE, EMBASE, and CENTRAL | STATA version 14 (StataCorp, College, TX) and Cochrane Review Manager 5.3 (London, UK). |
| **Mohiuddin et al, 2021** | No | No | No | No | The Cochrane Library, MEDLINE and Embase | R statistical software package |
| **Sahebally et al, 2021** | No | Only articles in English | At least 12-month follow-up. | No | PubMed, EMBASE, and Cochrane databases | Review Manager software (RevMan, version 5.3. Copenhagen: The Nordic Cochrane Centre, The Cochrane Collaboration, 2012) |
| **Prudhomme et al, 2021** | No | No | No | Yes | Ovid MEDLINE and WHO registry | General Package for Meta-Analysis ‘‘meta’’ Version 4.9-1 with R software version 3.5.1. |
| **Jones et al, 2018** | No | No | No | Yes | Cochrane Central Register for controlled trials, Ovid MEDLINE, EMBASE, Science citation index expanded | Review Manager 5 software to analyze the data (RevMan 2014). |
| **Findlay et al, 2018** | No | Only articles in English | No | No | PubMed, EMBASE and Cochrane Centre Register of Controlled Trials | RevMan v5.2 (The Cochran Collaboration); R v3.02 (R Core Team). |
| **Pianka et al, 2017** | No | No | No | No | PubMed, EMBASE and the Cochrane Library | R (Version 3.1.1, R Development Core Team 2015, Vienna, Austria). |
| **Cross et al, 2017** | No | No | No | No | Cochrane Central Register of Controlled Trials, MEDLINE, Embase and CINAHL | RevMan 5.3 (The Nordic Cochrane Centre, The Cochrane Collaboration, Copenhagen, Denmark). |
| **López-Cano et al, 2017** | No | English, Dutch, French, Polish, and Spanish | At least 12 month follow up | No | MEDLINE (PubMed), SCOPUS, CINAHL, Web of Knowledge, and EMBASE | RevMan 5.3 (The Nordic Cochrane Centre, The Cochrane Collaboration, Copenhagen, Denmark). |
| **Patel et al, 2017** | No | No | At least 12 month follow up | Yes | MEDLINE or EMBASE | RevMan 5.3 (The Nordic Cochrane Centre, The Cochrane Collaboration, Copenhagen, Denmark). |
| **Chapman et al, 2017** | No | No | At least 12 month follow up | No | MEDLINE, EMBASE and the Cochrane Library | RevMan 5.3 (The Nordic Cochrane Centre, The Cochrane Collaboration, Copenhagen, Denmark). |
| **Cornille et al, 2017** | No | No | No | No | PubMed, Embase™ and the Cochrane Library | RevMan 5.3 (The Nordic Cochrane Centre, The Cochrane Collaboration, Copenhagen, Denmark). |
| **Wang et al, 2016** | No | No | No | No | PubMed, Embase, and the Cochrane Library | Stata 12.0 (Stata Corporation, College Station, TX, USA); RevMan 5.3 (Nordic Cochrane Centre, Cochrane Collaboration, Copenhagen, Denmark). |
| **Zhu et al, 2016** | No | No | No | No | PubMed, EMBASE, Science Citation Index, and the Cochrane Libraries | RevMan 5.3 (The Nordic Cochrane Centre, The Cochrane Collaboration, Copenhagen, Denmark). |
| **Sajid et al, 2012** | No | No | No | No | MEDLINE, EMBASE, Science Citation Index, and the Cochrane Libraries | RevMan 5.1.2 (The Nordic Cochrane Centre, The Cochrane Collaboration, Copenhagen, Denmark). |
| **Shabbir et al, 2012** | No | No | No | No | MEDLINE, EMBASE and CENTRAL | RevMan 5 |
| **Wijeyekoon et al, 2010** | No | No | No | No | Cochrane Library trials register, Medline, Embase, Science Citation Index Expanded | RevMan 5.0 (The Nordic Cochrane Centre, The Cochrane Collaboration, Copenhagen, Denmark). |
| **Tam et al, 2010** | No | Only articles in English | No | No | MEDLINE, Embase, and Cochrane Library | Review Manager ver. 5 (Cochrane Collaboration, Oxford, UK). |

**Appendix Table 2.** Assessment of quality of the systematic reviews and the original studies included within

| **Review** | **Quality of the review** | **Quality of included studies** |
| --- | --- | --- |
| **Verdaguer-Tremolosa et al, 2023** | Low | 6 some concern and 2 high risk of bias |
| **McKechnie et al, 2022** | Critically low | 7 low and 2 high risk of bias |
| **Mohiuddin et al, 2021** | Low | 7 low, 1 high, 5 some concerns of bias |
| **Sahebally et al, 2021** | Low | Some concerns of bias in all studies |
| **Prudhomme et al, 2021** | Low | 1 low, 8 some concerns, 1 high risk of bias |
| **Jones et al, 2018** | Moderate | 6 low and 4 high risk of bias |
| **Findlay et al, 2018** | Low | Serious risk of bias |
| **Pianka et al, 2017** | Critically low | High risk of bias in all studies |
| **Cross et al, 2017** | Low | 5 low, 4 moderate, high risk of bias |
| **López-Cano et al, 2017** | Moderate | Low risk of bias in all studies |
| **Patel et al, 2017** | Low | 4 some risk and 5 high risk of bias |
| **Chapman et al, 2017** | Moderate | High risk of bias in all studies |
| **Cornille et al, 2017** | Low | High risk of bias in all studies |
| **Wang et al, 2016** | Low | 3 some concerns, 3 high risk of bias |
| **Zhu et al, 2016** | Low | 2 some concern, 6 high risk of bias |
| **Sajid et al, 2012** | Critically low | 2 low and 1 some concern of bias |
| **Shabbir et al, 2012** | Low | Good quality of all studies |
| **Wijeyekoon et al, 2010** | Moderate | High risk of bias in all studies |
| **Tam et al, 2010** | Critically low | 2 some concern, 1 high risk of bias |

**Appendix Table 3**. Assessment of the certainty of evidence for the main outcomes

|  | **Certainty assessment** | | | | | | | **Effect** | | **Certainty** |
| --- | --- | --- | --- | --- | --- | --- | --- | --- | --- | --- |
| **Outcome** | **№ of studies** | **Study design** | **Risk of bias** | **Inconsistency** | **Indirectness** | **Imprecision** | **Other considerations** | **Relative (95% CI)** | **Absolute (95% CI)** |  |
| **Clinically detected hernias** | 19 | Randomised trials | Serious^a^ | Serious^b^ | Not serious | Not serious | None | **HR 0.33** (0.26 to 0.41) | **0 fewer per 1,000** (from 0 fewer to 0 fewer) | ⨁⨁◯◯ Low |
| **Radiologically detected hernias** | 9 | Randomised trials | Serious^a^ | Serious^b^ | Not serious | Not serious | None | **HR 0.55** (0.45 to 0.68) | **1 fewer per 1,000** (from 1 fewer to 0 fewer) | ⨁⨁◯◯ Low |
| **Surgical repair of hernia** | 12 | Randomised trials | Serious^a^ | Serious^b^ | Not serious | Not serious | None | **HR 0.46** (0.35 to 0.62) | **0 fewer per 1,000** (from 1 fewer to 0 fewer) | ⨁⨁◯◯ Low |
| **Complications** | 9 | Randomised trials | Serious^a^ | Not serious | Not serious | Not serious | None | **HR 0.81** (0.66 to 1.00) | **1 fewer per 1,000** (from 1 fewer to 1 fewer) | ⨁⨁⨁◯ Moderate |

**CI:** confidence interval; **HR:** hazard Ratio

#### Explanations

a. Most studies had low quality in AMSTAR-2 assessment

b. The I^2^ index was between 25% and 75%
